# On the Alignment of Acoustic and Coupled Mechanic-Acoustic Eigenmodes in Phonation by Supraglottal Duct Variations

**DOI:** 10.3390/bioengineering10121369

**Published:** 2023-11-28

**Authors:** Florian Kraxberger, Christoph Näger, Marco Laudato, Elias Sundström, Stefan Becker, Mihai Mihaescu, Stefan Kniesburges, Stefan Schoder

**Affiliations:** 1 Institute of Fundamentals and Theory in Electrical Engineering (IGTE), Graz University of Technology, Inffeldgasse 18/I, 8010 Graz, Austria; stefan.schoder@tugraz.at; 2 Institute of Fluid Mechanics (LSTM), Friedrich-Alexander-Universität Erlangen-Nürnberg, Cauerstraße 4, 91058 Erlangen, Germany; christoph.naeger@fau.de (C.N.); stefan.becker@fau.de (S.B.); 3 Department of Engineering Mechanics, FLOW Research Center, KTH Royal Institute of Technology, Osquars Backe 18, 10044 Stockholm, Sweden; laudato@kth.se (M.L.); elias@kth.se (E.S.); mihai@mech.kth.se (M.M.); 4 Division of Phoniatrics and Pediatric Audiology, Department of Otorhinolaryngology, Head & Neck Surgery, Friedrich-Alexander-Universität Erlangen-Nürnberg, Waldstraße 1, 91054 Erlangen, Germany; stefan.kniesburges@uk-erlangen.de

**Keywords:** voice production, fluid-structure-acoustic interaction, mechanical-acoustical eigenvalue simulation, vocal fold motion, finite element model

## Abstract

Sound generation in human phonation and the underlying fluid–structure–acoustic interaction that describes the sound production mechanism are not fully understood. A previous experimental study, with a silicone made vocal fold model connected to a straight vocal tract pipe of fixed length, showed that vibroacoustic coupling can cause a deviation in the vocal fold vibration frequency. This occurred when the fundamental frequency of the vocal fold motion was close to the lowest acoustic resonance frequency of the pipe. What is not fully understood is how the vibroacoustic coupling is influenced by a varying vocal tract length. Presuming that this effect is a pure coupling of the acoustical effects, a numerical simulation model is established based on the computation of the mechanical-acoustic eigenvalue. With varying pipe lengths, the lowest acoustic resonance frequency was adjusted in the experiments and so in the simulation setup. In doing so, the evolution of the vocal folds’ coupled eigenvalues and eigenmodes is investigated, which confirms the experimental findings. Finally, it was shown that for normal phonation conditions, the mechanical mode is the most efficient vibration pattern whenever the acoustic resonance of the pipe (lowest formant) is far away from the vocal folds’ vibration frequency. Whenever the lowest formant is slightly lower than the mechanical vocal fold eigenfrequency, the coupled vocal fold motion pattern at the formant frequency dominates.

## 1. Introduction

The human voice is physically created in a complex process characterized by fluid-structure-acoustic interaction [1]. In this process, the vocal folds are excited to vibrate by the airflow of the lungs $\dot{V}$. During vocal fold vibration, the superficial tissue of the vocal fold moves in a wave-like manner, exhibiting a vertical phase difference. This motion generates what is known as a “mucosal wave”, with a frequency that plays a role in determining the pitch of the voice. This vibration leads to a modulation of the airflow, forming a pulsating free jet in the vocal tract. The sound that constitutes the voice thereby arises aero-acoustically from the turbulent free jet region [2,3,4], as well as vibro-acoustically by sound radiation from the vibrating vocal fold surface [5]. This sound is filtered by the vocal tract and radiated through the mouth and nares, resulting in the voice pattern. A linear behavior between the sound source and filter was assumed for a long time, i.e., changes in the source due to the filter were neglected [6]. However, this simplified representation is not always valid, especially when a resonance frequency of the vocal tract or the trachea region $f_{R}$ is close to the vibration frequency of the vocal folds $f_{o}$ [7]. This behavior was also observed in a patient study recently [8]. It has been studied using a lumped-mass description of tissue mechanics, quasi-steady flow, and one-dimensional acoustics in [9]. In doing so, frequency deviations and maxima jump of the threshold pressure occur when the mechanical oscillation frequency is slightly above a vocal tract resonance. Both the trachea and the vocal tract may produce those same effects [10,11]. In [12], the assumption that vocal tract formants interact with the voice source was analyzed in vivo by investigating the data collected by a study consisting of twelve classical singers. The analysis used transnasal high-speed videoendoscopy, electroglottography, and audio recordings. However, the presented data partially corroborates that vowel transitions may result in level-two interactions (using the nomenclature of Titze [7]). The authors of [13] reported that under certain conditions, e.g., singing voice, the fundamental frequency of the vocal folds can go up and interfere with the formant frequencies. So, acoustic feedback from the vocal tract filter to the vocal fold motion becomes strong and non-negligible. Again, a multi-mass model was used to confirm the findings [9]. Due to the complexity of the problem, often only simple metrics such as the variation of $f_{0}$ or the change in the threshold of transglottal pressure for oscillation have been studied. Therefore, in [14,15], first experimental measurements are conducted to gain further insight into the interaction between vocal tract acoustics, structural dynamics, and aerodynamics for different vocal tract lengths. The variation of the vocal tract length thereby changes the acoustic properties of the vocal tract, allowing a systematic investigation of the relationship between flow and acoustics.

Within the present contribution, the experimental results reported in [14,15] are extended substantially, and accompanying simulations are conducted to report the details on the non-negligible mechanical-acoustic back-coupling (feedback of the vocal tract resonances on the vibration) which is leading to the so-called “non-linear” filtering property at certain conditions (e.g., singing). Thereby, nonlinear effects can be, e.g., a nonlinear behavior of the vocal fold material, large mechanical deformations, or a contact between the vocal folds during phonation. For this purpose, experimental investigations will be performed on a simplified, synthetic larynx model using laser scanning vibrometry and high-speed camera (HSC) records. From a computational perspective, the simplified lumped-mass representation and the one-dimensional wave equation presented in [9] is significantly extended to a three-dimensional finite element model, being able to represent any given upper airway geometry [16]. This numerical simulation model is able to describe the most efficient vocal fold motion mode and the phenomena of the linear filter range, as well as the non-linear interaction and coupling of modes potentially leading to deviations in the oscillation frequency and the maxima jumps. Nevertheless, the governing equations of the acoustic and structural dynamics fields are linear, meaning that no hard contact between the vocal folds or nonlinear mechanical material laws is incorporated. Furthermore, the similarity of these numerically computed modes with experimental data is used to explain the details of the mechanical-acoustic feedback. The findings exhibit that the presented simulation approach yields similar results to the measurements which enables to use the simplified linear numerical model to gain insights into the linear coupling effects between mechanic and acoustic fields. In the final discussion, the limitations are explained and the connection of the numerical and experimental findings to other voice parameters like the sound pressure level and vocal efficiency are drawn.

The paper is organized as follows. Section 2 describes the experimental setup. In Section 3, the numerical model of the quadratic mechanical-acoustic eigenvalue system is presented. Section 4 reports the experimental results of the vocal fold motion and the numerical results of the eigenmode analysis. The application results are discussed in Section 5 providing also the model limitations. Conclusions are drawn in Section 6.

## 2. Experimental Study

**Figure 1:**
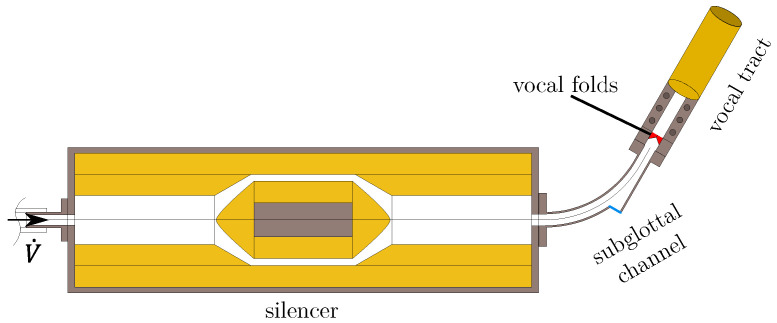
The experimental setup. The vocal folds are marked in red and are located between the vocal tract and the curved subglottal channel. A silencer is placed in front of the vocal folds to dampen the sound in the inflow. The flow direction is from left to right.

The synthetic vocal folds are based on the vocal fold M5 geometry model, proposed by Scherer et al. [17], with the detailed geometry presented in [14] and were cast from a single layer of silicone. The specimens were made of a three-component addition-cure silicone (Smooth-on, Inc., Macungie, PA, USA). The compound consists of a two-part Ecoflex 0030^©^ (A + B) silicone rubber and three-parts silicone thinner (T) assembling the mixture 113 as described in [18]. The experimental test rig is shown schematically in Figure 1. The flow through the setup with a volume discharge of $\dot{V}$ is generated by a mass flow generator [19] and takes place from left to right as drawn by the arrow at the inflow. First, the flow is acoustically preconditioned by an acoustic silencer [20,21] before entering the subglottal tube and then the vocal folds. The vocal folds are marked in red and are located between the subglottal channel and the first section of the simplified vocal tract, both having a rectangular cross-section of Δy×Δx=18mm×15mm that anatomically corresponds to the lateral–longitudinal orientation of a human larynx. The vocal tract consists of two sections: the first is a rectangular channel with the same dimensions as the vocal folds and the subglottal channel. Connected to this, there is a second section that has a circular cross-section. The second section consists of two telescopic tubes that enable continuously varying the length of the vocal tract, allowing its acoustic resonance frequencies to be adjusted. The measurements are performed for different vocal tract lengths *L* in the range L∈[170,930]mm.

Additionally, the vocal fold motion was investigated by laser scanning vibrometry (LSV) in [14]. Before the vocal folds section, a curved subglottal channel is placed with a small optical window to record the vocal folds’ surface motion (see Figure 1).

**Figure 2:**
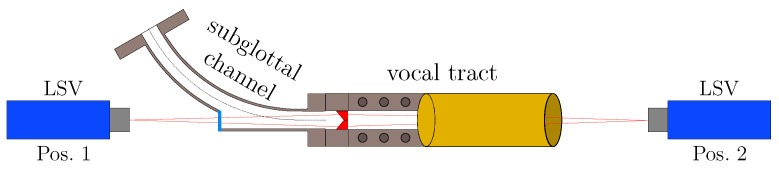
Schematic of the experimental setup for the LSV measurements.

This approach can examine the surface motion on the subglottal and supraglottal sides using two LSVs. As depicted in Figure 2, the LSV measurement positioning is illustrated. A total of 748 measurement points are measured, 374 on each vocal fold surface. For the high-speed camera recordings, the camera is set up at position 2 to record the surface motion by video. A wall pressure sensor is placed in the subglottal channel below the vocal folds to synchronize the measurements at a specific start time of each measurement. Its signal generates a trigger signal that determines the start time.

## 3. Numerical Model

This section describes the numerical model using the Finite Element (FE) method. All numerical simulations have been performed using *Ansys Mechanical 2022 R2* [22,23].

### 3.1. Governing Equations

The governing equations of the acoustic and solid mechanic field, as well as their physical coupling conditions, are discussed in the following.

#### 3.1.1. Acoustic Field

**Figure 3:**
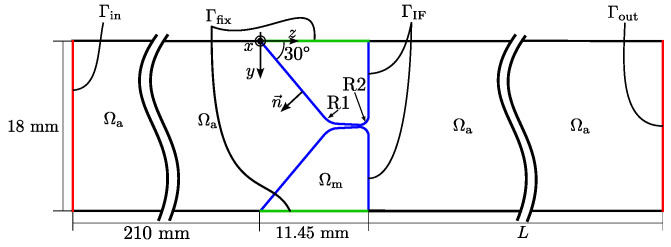
Schematic sketch of the longitudinal cut of the investigated geometry. $\Omega_{a}$ and $\Omega_{m}$ are the acoustic and mechanic domains, respectively, $\Gamma_{\mathrm{IF}}$ is the interface surface (blue), $\Gamma_{\mathrm{fix}}$ is the fixed surface of the mechanic domain (green), and $\Gamma_{\mathrm{in}}$ and $\Gamma_{\mathrm{out}}$ are inlet and outlet surfaces (red), respectively. The length *L* is varied.

Using the linear acoustic wave equation [24] (Equation (5.28)) to describe the acoustic field in the context of human phonation is state of the art and this approach is commonly used in the literature, e.g., [1,3,16,25]. Therefore, the acoustic field in a 3D acoustic domain $\Omega_{a}$ is governed by

(1) $$\frac{1}{c_{0}^{2}}\frac{\partial^{2}p}{\partial t^{2}}-\Delta p=0,$$

where *p* is the acoustic pressure, $c_{0}=\sqrt{K/\rho_{0}}$ is the isentropic speed of sound computed from the bulk modulus *K*, and the ambient fluid mass density $\rho_{0}$, *t* is time, and Δ=∇·∇ is the Laplacian. For the acoustic domain, all boundaries except the inlet and outlet surfaces $\Gamma_{\mathrm{in}}$ and $\Gamma_{\mathrm{out}}$, and the interface boundary to the mechanic domain $\Gamma_{\mathrm{IF}}$, as depicted in Figure 3 are considered sound hard, i.e., homogeneous Neumann boundary conditions are imposed on the pressure. This is justified by the fact that the specific acoustic impedance of air (a fluid) is several orders of magnitude smaller than the specific acoustic impedance of the duct wall of the experimental setup made of acrylic glass, and the large impedance jump of three orders of magnitude, is approximated by a sound-hard boundary condition. At the surface $\Gamma_{\mathrm{in}}$, a homogeneous Dirichlet boundary condition is applied on the pressure, which models a sound soft boundary [24] (Chapter 5.4). At $\Gamma_{\mathrm{out}}$, the radiation impedance is adjusted to the test rig [26]. For air at 22 ${}^{\circ }C$ (experimental condition), which is the medium in the acoustic domain $\Omega_{a}$, typical values are $c_{0}=346.25\;m/s$ and $\rho_{0}=1.225\;kg/m^{3}$.

#### 3.1.2. Mechanic Field

Newton’s second law (conservation of momentum) in differential form states that

(2) $$\vec{f}=\rho \frac{\partial^{2}\vec{d}}{\partial t^{2}},$$

where $\vec{f}=\nabla \;\cdot \;\sigma +\vec{g}$ is the force density computed from the stress tensor σ and external body forces $\vec{g}$, ρ is the material’s mass density, and $\vec{d}=\left(d_{1},d_{2},d_{3}\right)$ is the displacement. Thus, Equation (2) can be reformulated to

(3) $$\rho \frac{\partial^{2}\vec{d}}{\partial t^{2}}-\nabla \;\cdot \;\sigma =\vec{g}.$$

The external body forces are assumed to be zero. Furthermore, a linear elastic stress strain s relationship is assumed σ=C:s in concludence with [27], where the stiffness tensor C depends on the Young’s modulus *E* and the Poisson ratio ν, as defined in [23] (Equations (2)–(4)). The silicone rubber material used in the experiments was characterized in [18], called “mixture 113”. From [18] (Table 3), we know that for the used silicone mixture ν=0.499 and $\rho =976\;kg/m^{3}$. The Young’s modulus *E* will be described in Section 3.3.

#### 3.1.3. Finite Element Formulation of Mechanic-Acoustic Coupling

Using the FE method, the following matrix-vector equation is set up for each finite element, of the coupled mechanic-acoustic problem [23] (Equations (8)–(32))

(4) $$\left[\begin{array}{cc} M_{e} & 0\\ M^{\mathrm{fs}} & M_{e}^{p} \end{array}\right]\frac{\partial^{2}}{\partial t^{2}}\left(\begin{array}{c} d_{e}\\ p_{e} \end{array}\right)+\left[\begin{array}{cc} C_{e} & 0\\ 0 & C_{e}^{p} \end{array}\right]\frac{\partial }{\partial t}\left(\begin{array}{c} d_{e}\\ p_{e} \end{array}\right)+\left[\begin{array}{cc} K_{e} & K^{\mathrm{fs}}\\ 0 & K_{e}^{p} \end{array}\right]\left(\begin{array}{c} d_{e}\\ p_{e} \end{array}\right)=\left[\begin{array}{c} F_{e}\\ 0 \end{array}\right],$$

with $d_{e}$ and $p_{e}$, being mathematical vectors collecting the unknowns at the degrees of freedom from one element, for (mechanic) displacement and (acoustic) pressure, respectively. The size of $d_{e}$ is $3N_{\mathrm{dof}},e$ and of $p_{e}$ it is $N_{\mathrm{dof}},e$, where $N_{\mathrm{dof}},e$ is the number of degrees of freedom. Considering one node of the mesh, either 3 degrees of freedom for the displacement or one degree of freedom for the pressure are sought, depending on the location of the respective node in the computational domain. Thereby, $M_{e}$, $C_{e}$ and $K_{e}$ are standard element mass, damping and stiffness matrices of the mechanics domain, respectively [23] (Section 2.2). Furthermore, $M_{e}^{p}$, $C_{e}^{p}$, and $K_{e}^{p}$, are the standard element mass, damping and stiffness matrices of the acoustic domain [23] (Section 8.2). $C_{e}^{p}$ accounts for the damping of the open duct radiation [26]. The coupling terms are $M^{\mathrm{fs}}$ for the kinematic coupling condition and $K^{\mathrm{fs}}$ for the dynamic coupling condition, which are described in [23] (Section 8.4). At the interface between air and the vocal folds, the continuity requires that the normal component of the mechanical surface velocity is equal to the normal component of the acoustic particle velocity. This is the so-called kinematic coupling condition and can be rewritten in an acoustic pressure formulation as [24] (Section 8.1)

(5) $$\vec{n}\;\cdot \;\left(\frac{\partial^{2}\vec{d}}{\partial t^{2}}+\frac{1}{\rho_{0}}\nabla p\right)=0,$$

where $\vec{n}$ is the outward pointing normal vector at the interface $\Gamma_{\mathrm{IF}}$, which is depicted in Figure 3. Furthermore, the continuity of the normal component of the forces must be satisfied [24] (Section 8.1), i.e.,

(6) $$\vec{n}\;\cdot \;\sigma +\vec{n}p=0.$$

Finally, the forcing vector $F_{e}$ is zero due to no externally applied element forces.

### 3.2. Eigenvalue Problem

In order to recast the system of Equation (4) into an eigenvalue problem, a harmonic ansatz for pressure and displacement is introduced as

(7) $$p=R\;\{\tilde{p}e^{i\omega t}\},\;\vec{d}=R\;\left\{\tilde{\vec{d}}e^{i\omega t}\right\},$$

where ω=2πf is the angular frequency and “i” is the imaginary unit. Therewith, using zero forcing on the right-hand side of the system of equations, Equation (4) is reformulated to

(8) $$-\omega^{2}\left[\begin{array}{cc} M_{e} & 0\\ M^{\mathrm{fs}} & M_{e}^{p} \end{array}\right]\left(\begin{array}{c} {\tilde{d}}_{e}\\ {\tilde{p}}_{e} \end{array}\right)+i\omega \left[\begin{array}{cc} C_{e} & 0\\ 0 & C_{e}^{p} \end{array}\right]\left(\begin{array}{c} \tilde{d}\\ {\tilde{p}}_{e} \end{array}\right)+\left[\begin{array}{cc} K_{e} & K^{\mathrm{fs}}\\ 0 & K_{e}^{p} \end{array}\right]\left(\begin{array}{c} {\tilde{d}}_{e}\\ {\tilde{p}}_{e} \end{array}\right)=\left[\begin{array}{c} 0\\ 0 \end{array}\right],$$

which is a quadratic eigenvalue problem in ω. Using the substitution method described in [23] (Section 15.16.6), the quadratic eigenvalue problem is transformed into an equivalent system of generalized eigenvalue problems. This system is solved with *Ansys Mechanical* [22].

### 3.3. Material Damping Model

Rupitsch et al. [18] characterized the damping behavior of the same material used for the present study. Therein, they introduced a model for a complex-valued frequency-dependent equivalent Young’s modulus E(f) as

(9) $$\begin{array}{cc} E(f) & =E_{R}\left(f\right)+iE_{I}\left(f\right),\;\tan\delta (f)=2\xi \left(f\right)=\frac{E_{I}\left(f\right)}{E_{R}\left(f\right)},\\ E_{R}\left(f\right) & =A_{R}+B_{R}f+C_{R}\log_{10}\;\left(\frac{f+1\mathrm{Hz}}{1\mathrm{Hz}}\right),\\ E_{I}\left(f\right) & =A_{I}+B_{I}f+C_{I}\log_{10}\;\left(\frac{f+1\mathrm{Hz}}{1\mathrm{Hz}}\right), \end{array}$$

where $\left\{A_{R},B_{R},C_{R}\right\}$ are coefficients of the real part $E_{R}\left(f\right)$, and $\left\{A_{I},B_{I},C_{I}\right\}$ are coefficients of the imaginary part $E_{I}\left(f\right)$ of E(f). Thereby, $A_{R}$ is equivalent to the static Young’s modulus, which can be obtained by a tensile test. From real and imaginary parts $E_{R}\left(f\right)$ and $E_{I}\left(f\right)$, the damping ξ(f) and a loss factor tanδ(f) can be obtained. For the silicone mixture material of interest, Rupitsch et al. [18] found parameters for E(f), as listed in Table 1, by fitting parameters of a FE simulation to measurements results from a vibration transmission analyzer.

The model of Rupitsch et al. [18] can be interpreted as an extension to the standard Rayleigh damping model [24] (Section 3.7.2). However, Rupitsch’s model is not available in Ansys Mechanical; therefore, the material model introduced in [18] is approximated in an $f_{1}=140\mathrm{Hz}$ with a Rayleigh damping model. Note, that this Rayleigh damping model is only an approximation. Using the model of Rupitsch et al. [18] would lead to a non-linear eigenvalue simulation [28] instead of the linear one proposed, limiting the applicability of the model to frequencies around $f_{1}=140\mathrm{Hz}$. However, the iterative algorithm increases the computational cost significantly, and therefore a simple Rayleigh approximation of Rupitsch’s model has been found as a suitable tradeoff balancing computational cost and accuracy for the present investigation. Thus, the damping matrix $C_{e}$ in the mechanic domain in Equation (8) is composed of a linear combination of mass and stiffness matrices

(10) $$C_{e}=\alpha M_{e}+\beta K_{e}.$$

The Rayleigh model coefficients α and β are obtained from ξ(f) and $E_{R}\left(f\right)$ at a frequency of interest $f_{1}$ using a small frequency deviation Δf [24] (p. 113), such that

(11) $$\begin{array}{cc} \beta (f_{1}) & =\frac{4\Delta f\xi (f_{1})}{{\left(f_{1}+\Delta f\right)}^{2}-{\left(f_{1}-\Delta f\right)}^{2}}\\ \alpha (f_{1}) & =2\left(f_{1}+\Delta f\right)\xi \left(f_{1}\right)-\beta {\left(f_{1}+\Delta f\right)}^{2} \end{array}$$

In Table 2, the obtained values for $\alpha (f_{1})$ and $\beta (f_{1})$ and Young’s modulus $E_{R}\left(f_{1}\right)$ are listed for the operating frequency $f_{1}=140\;\mathrm{Hz}$.

**Figure 4:**
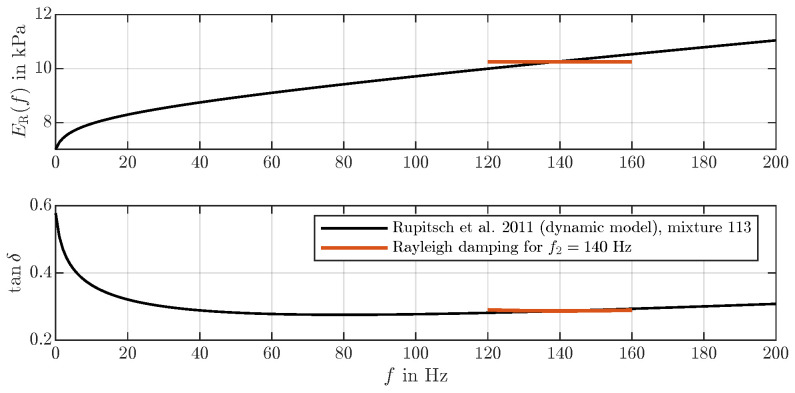
Comparison of the dynamic model of [18] with Rayleigh damping in frequency bands of ±20Hz around the operating point $f_{1}=140\mathrm{Hz}$.

The Rayleigh approximation of the Rupitsch model will use a frequency-independent Youngs modulus. In Figure 4, the model of Rupitsch et al. is compared to the standard Rayleigh damping model in the operating point $f_{1}=140\;\mathrm{Hz}$ exemplarily. For further calculations, an operating point in the experimentally estimated fundamental frequency $f_{0}$ range is selected [14].

### 3.4. Simulation Setup

In Figure 3, a sketch of the longitudinal cut of the investigated 3D-geometry is depicted. It shows the acoustic and mechanic domains $\Omega_{a}$ and $\Omega_{m}$, respectively. For the numerical simulations, the length *L* is varied from 200 mm to 900 mm in steps of 20 mm, see Figure 3.

### 3.5. Mesh Convergence Study

The convergence of the FE model with decreasing element size (using second-order finite elements) was assessed. In a similar manner as in [29], a relative frequency error $Err_{\mathrm{rel},f}^{L_{2}}$,

(12) $$Err_{\mathrm{rel},f}^{L_{2}}=\sqrt{\frac{\sum_{i=1}^{N_{\mathrm{modes}}}{\left(f_{\mathrm{mode},\mathrm{ref}}^{\left(i\right)}-f_{\mathrm{mode},\mathrm{num}}^{\left(i\right)}\right)}^{2}}{\sum_{i=1}^{N_{\mathrm{modes}}}{\left(f_{\mathrm{mode},\mathrm{ref}}^{\left(i\right)}\right)}^{2}}}\;$$

was defined, where $f_{\mathrm{mode},\mathrm{ref}}^{\left(i\right)}$ are the modal frequencies of a reference (benchmark) simulation using a very fine mesh, and $f_{\mathrm{mode},\mathrm{num}}^{\left(i\right)}$ are the modal frequencies of the mesh that is tested against the reference. The number of investigated modes is $N_{\mathrm{modes}}=20$ accounting for the range of interest. The parameters of the investigated meshes and the result of the mesh convergence study are listed in Table 3. The approximate element size has been chosen based on an upper frequency limit of 8.5kHz, resulting in a wavelength λ that is discretized with 10, 20, or 40 elements in the subglottal and supraglottal regions (also called “duct” in Table 3). The region around the vocal folds is discretized much finer and unstructured due to the small glottal gap that must be discretized sufficiently fine. For the later numerical study, mesh 2 of Table 3 was used, as an error $Err_{\mathrm{rel},f}^{L_{2}}<$ 0.5% was seen as an acceptable compromise between computational speed and accuracy. The discretization error reduces monotonically. Approximately, it reduces by two orders of magnitude for each grid refinement order. This correlates with the use of a second-order spatial discretization schemes.

## 4. Results

### 4.1. Experimental Results

**Figure 5:**
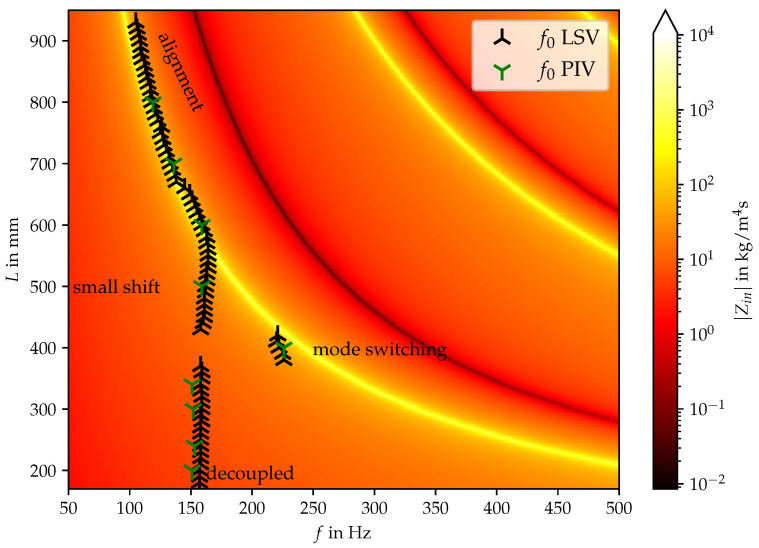
Vocal tract input impedance as a function of frequency *f* and length *L*. Superimposed are the primary oscillation frequencies of the vocal folds $f_{o}$ for the individual measurements.

From the LSV results of the fluid flow perturbation, the oscillation frequency of the vocal folds can be estimated using the discrete Fourier transform. The measurements were conducted in the range L∈[170,930]mm with an increment of 10 mm between the measurements. The results of this analysis, i.e., the primary oscillation frequency of the vocal folds $f_{0}$ as a function of the duct length, are reported in Figure 5. The background of this figure is colored by the acoustic input impedance of the vocal tract computed via a transmission line model [30]. For this purpose, the VT is divided into sections of equal cross-section, whereby the frequency-dependent vocal tract input impedance $Z_{\mathrm{in}}$ can be calculated by a concatenation of matrix multiplications. The maxima of the reactance of $Z_{\mathrm{in}\;}$ correspond to the VT resonance frequencies [26], which coincides for the current duct configuration with the maximum of the amplitude of $Z_{\mathrm{in}}$. Additionally, the fundamental frequency $f_{0}$ based on particle image velocimetry data from [31] is displayed in Figure 5. In doing so, the time series signals are Fourier transformed and the most dominant peak in the amplitude spectrum is picked defining the respective $f_{0}$ at a corresponding duct length *L*. Figure 5 shows clearly that the trends of the experiments are reproducible. For duct length below L=360 mm, a regime is detected where the acoustic resonance of the duct does not influence the fundamental frequency $f_{0}$. At around L=400 mm, the oscillation shape of the vocal folds is suddenly completely different explained by mode switching of the silicon vocal fold oscillation. In a study by Sundberg et al. [8], pitch jumps and voice breaks occured for singers through long tube with different resistances [8]. For larger lengths and below the actual alignment with the acoustic resonance frequency of the duct, the fundamental frequency is gradually shifted towards the acoustic resonance frequency with increasing duct length until a length of about L=600 mm. Above this duct length, alignment takes place with the acoustic resonance frequency of the duct.

**Figure 6:**
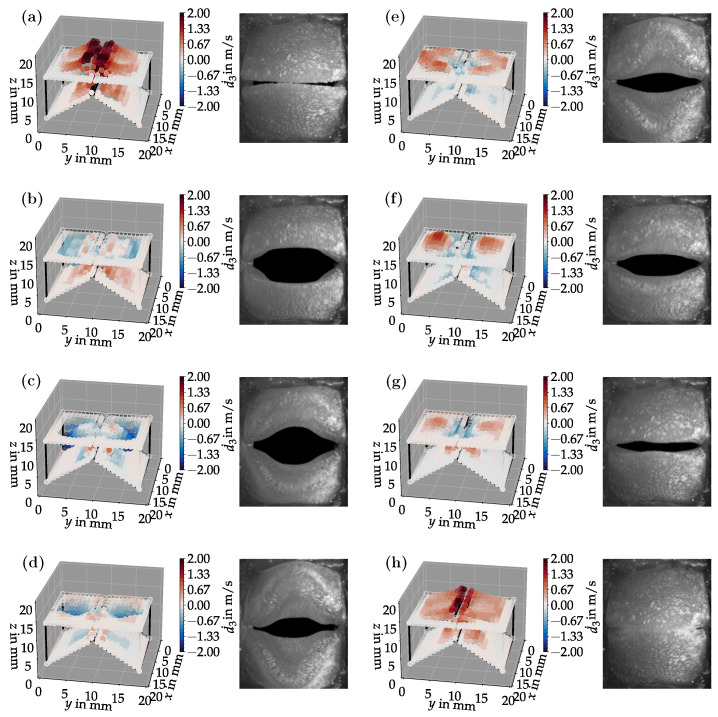
Time-synchronized LSV surface velocity and HSC measurements for a duct length of L=200 mm. The deflection of the points as well as the color in the LSV plots are proportional to the measured surface velocity. The sequence (**a**–**h**) shows the oscillation of one complete period.

Figure 6a–h shows eight snapshots from the synchronized LSV and HSC measurements for a duct length of L=200 mm. The LSV measurement points were phase-synchronized based on a subglottal pressure sensor signal. The sensor recorded the $f_{0}$-periodic pressure time series from which the LSV records were triggered. These are equidistantly distributed over one vibration period. The surface velocities are in the range of about 2m/s. It can be seen well that during the opening process (Figure 6a), the velocities are maximum in the positive direction. This can be explained by the fact that before this, in the closed state, high subglottal pressure quickly builds up upstream of the vocal folds, accelerating the vocal folds upward while simultaneously opening them. The HSC images also show a clear convergent-divergent deformation of the glottis. While in images (a) and (b), there is still a convergent glottal duct shape, in image (c), the tips of the vocal folds begin to oscillate toward the upper and lower edges, respectively, so that in images (d) and (e), a divergent glottal duct shape can be seen. From image (f), a reverse process occurs again to the initial position (a). This convergent-divergent glottal duct deformation is essential to phonation to efficiently drive self-sustaining vocal fold oscillations [32].

**Figure 7:**
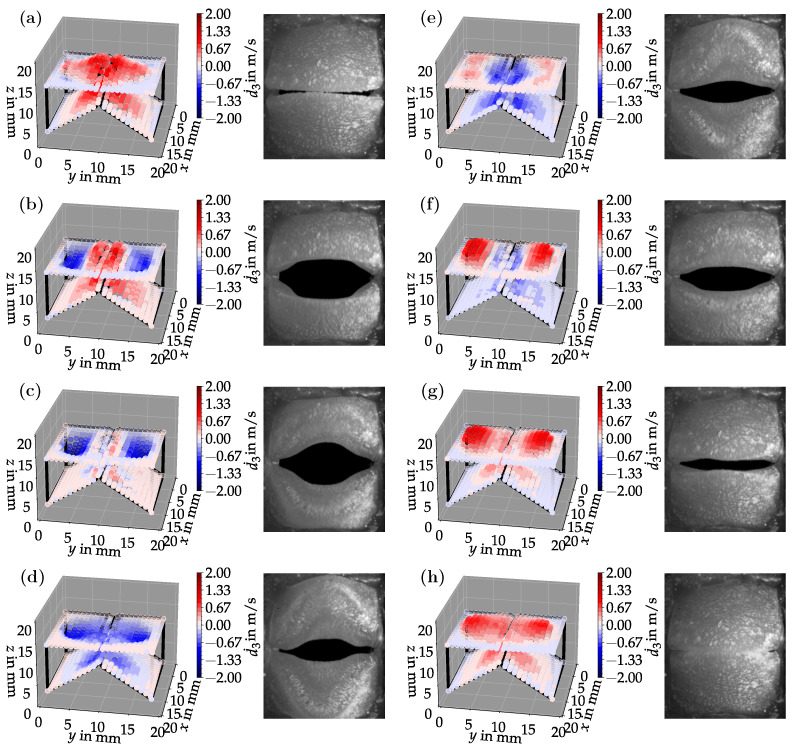
Bandpass filtered LSV data synchronized with the HSC recordings for a duct length of L=200 mm. The oscillation at the fundamental frequency (1. mode) can be seen. The sequence (**a**–**h**) shows the oscillation of a complete period.

Bandpass filtering can extract individual oscillation modes from the LSV data. The result of such filtering with cut-on and cut-off frequencies of 130Hz and 180Hz, respectively, can be seen in Figure 7.

**Figure 8:**
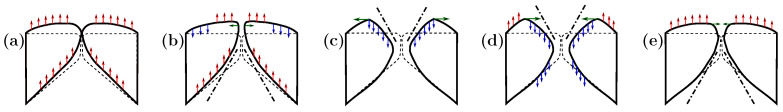
Schematic representation of vocal fold movement. The schemas (**a**–**e**) show different timesteps of one oscillation cycle. The red and blue arrows indicate the direction of surface velocity, and the green arrows indicate the direction of motion as revealed by the HSC images. The changing surface velocities on the vocal fold top cause rotation, responsible for the convergent-divergent glottal motion.

The filter has isolated the lowest oscillation frequency $f_{0}$ in the surface velocity spectrum, illustrating the oscillation at this first peak. Figure 8 shows a schematic representation of the motion in this lowest oscillation mode. It can be seen that immediately after the opening of the glottis, the surface velocities on the vocal fold top change from positive to negative direction (Figure 7a–c). Figure 8 summarizes, in (a)–(c), the opening of the vocal folds and illustrates the surface velocities accordingly. This leads to a rotation of the upper surface, changing the convergent glottis shape to a divergent shape. As soon as the maximum angle of divergence is reached, the opposite effect takes place, causing counter-rotation and a convergent glottis shape again (Figure 7d–f) and in Figure 8 illustrations (c)–(e). It can be concluded that the first mode is crucial for the divergent-convergent nature of vocal fold oscillation. Thus, it is an important component of self-sustained oscillation.

### 4.2. Numerical Results

Using the FE-based eigenvalue solver provided in Ansys Mechanical [22], the eigenvalues ω have been evaluated for (i) the acoustic system and (ii) the coupled mechanic-acoustic system. For the coupled mechanic-acoustic systems, the viscoelastic material at an operating condition of $f_{1}=140\mathrm{Hz}$ was used, as described in Section 3.3. The radiation impedance of the open end was modeled such that the fundamental frequencies of the acoustic modes match the test rig ones.

In Table 4, the first ten mode shapes from the numerical simulations are visualized for four duct lengths L={200,400,700,800}mm. A view analogous to LSV Pos. 2 and HSC (see Figure 2) has been used, which shows the superior surface view of the deformed VFs superimposed by a color proportional to the displacement in *z*-direction $d_{3}$.

At L=200mm, mode index 1 represents a mechanical mode opening and closing the vocal folds with in-phase displacement in *z*-direction at a frequency of f=81.29Hz. This mode possibly provides both aerodynamic constriction of the airflow as well as an effective coupling to the acoustic plane wave with its in-phase displacement. The same is valid for L∈{400 mm,700 mm,800 mm}. Mode index 2, which is at f=81.98Hz for L=200 mm does not contribute to acoustic radiation, because the *z*-component of the displacement is not in phase for both vocal folds hence no acoustic wave is scattered to the far field. The same is valid for L∈{400 mm,700 mm,800 mm}. At L=200 mm, mode index 3 represents an acoustically ineffective mechanical mode due to the displacement being out of phase within the vocal folds for a frequency of f=125.19Hz. The same holds for L∈{400 mm,700 mm}. However, at L=800 mm, an additional coupled mechanical-acoustic motion pattern arises, which is similar to mode index 1. Based on the mode shape morphology with a symmetric phase of the displacement *z*-component (where the symmetry axis is the glottis center line), this mode is acoustically effective. For mode index 4 at L=200 mm being at f=125.24Hz the asymmetric displacement *z*-component suggests an acoustically non-efficient mechanical mode. The same is valid for L∈{400 mm,700 mm}. Due to the additional coupled mechanical-acoustic motion pattern at L=800 mm however, mode index 4 of this length is the same as mode index 3 for L=700 mm, which is again acoustically ineffective. Mode index 5 at L=200 mm presents an acoustically effective vibration pattern at a frequency of f=137.88Hz due to the symmetry of the displacement *z*-component. Identical vibration patterns are present at L=400 mm and L=700 mm, where in the latter case only a sign change is present overall. In contrast to that, mode index 5 at L=200 mm at f=124.60Hz is acoustically ineffective due to the asymmetric displacement *z*-component. For mode index 6 at L=200 mm being at f=138.06Hz, as well as mode index 6 at L=400 mm, acoustically ineffective vibration patterns are present. In contrast to that, at L=700 mm an acoustically effective additional coupled mechanical-acoustic motion pattern arises, which is visually very similar to mode index 3 at L=800 mm. Furthermore, at L=800 mm, an acoustically effective mode is present. Mode indices 7 and 8 present acoustically ineffective vocal fold vibration patterns for all four VT length configurations. For mode index 9 at L=200 mm, which is at a frequency of f=154.04Hz, an acoustically effective motion pattern is present. However, for L∈{400 mm,700 mm,800 mm}, the modes are acoustically ineffective due to the asymmetries in the displacement *z*-component. Finally, for mode index 10 at L=200 mm being at f=154.23Hz, an acoustically inefficient mode is present. The same holds for L=400 mm. However, at L∈{700 mm,800 mm}, the *z*-component of the displacement is symmetric at the glottis center line, indicating acoustically effective vocal fold vibration modes at these length configurations.

Summarizing the above, from Table 4 it is evident that the first ten mode shapes do not change significantly comparing the cases L=200 mm and L=400 mm. However, at L=700 mm, an additional mode shape arises at mode 6, which shifts the subsequent mode indices. The same additional mode shape also arises in the case of L=800 mm, which is mode 3, resulting once more in a shift of the mode indices. Regarding the effective contributions of individual modes to phonation, it is important that the top surfaces of both VFs have the same phase throughout. Therefore, for the cases L=200 mm and L=400 mm, the following modes can be considered effective for phonation: Modes 1, 5, and 9, with other modes having only minor contributions to phonation. The other modes 2, 3, 4, 6, 7, 8, 10 are counterbalancing displacements and velocity patterns. This counterbalancing pattern is not able to couple efficiently to a plane acoustic wave inside the acoustic duct, which is in the case of the lowest formant a requirement for an effective coupling of the mechanical and acoustic field. For instance, mode 2 is a parallel motion of the left and right vocal fold which does not constrict the gap between the vocal folds and thus being a very inefficient mode from the whole fluid-structure-acoustic interaction perspective. This behavior in a higher order shape occurs for mode 4, 6, and 8. For the case L=700 mm, the following modes can be considered effective for phonation: Modes 1, 5, 6, 10. Finally, for case L=800 mm, the following modes can be considered effective for phonation: Modes 1, 3, 6, 10. It can be clearly seen, that in the case of L=700 mm and L=800 mm an additional mode (“phonation-effective mode”) enters being the mode shape with mode number 6 and 3 respectively. This phonation-effective mode is the coupled mechanical-acoustic motion pattern when the acoustic back-coupling is active. When this mode occurs, the mode numbers above this additional mode are shifted by one k←k+1. This behavior can be seen that the mode 5 in the case of L=700 mm is mode 6 for the case of L=800 mm.

**Figure 9:**
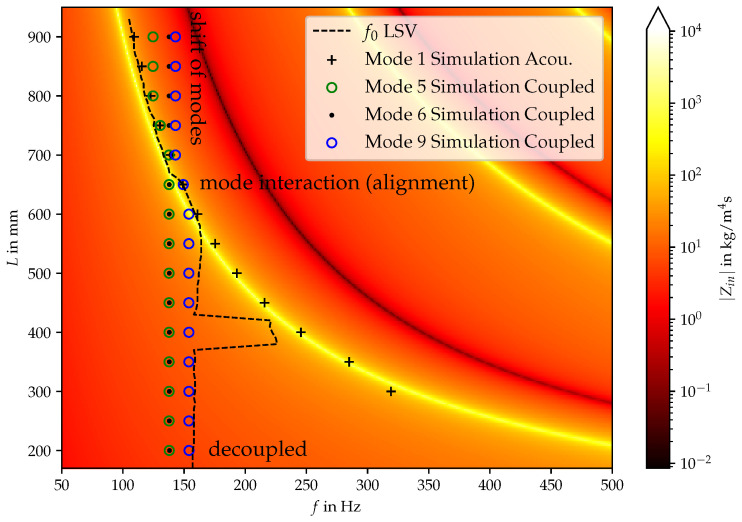
Numerically evaluated eigenfrequencies of the acoustic and coupled mechanic-acoustic system. The mechanical damping has been modeled by Rayleigh damping, which approximates the Rupitsch model at the operating frequency of f=140Hz.

The additional modes are further investigated by connecting them with the acoustic impedance of the vocal tract in the following. Based on visualizations of the mechanic displacement, modes have been identified that are similar to those of the experimental investigations. Figure 9 supplements Figure 5 by adding the simulation results of the linear eigenfrequency (mode index 5, 6 and 9) of the coupled mechanic-acoustic eigenmode simulation for varying duct length. For a duct length of less than 550 mm, the acoustic and the mechanical eigenmodes are decoupled from each other, suggesting that acoustic effects do not significantly influence the silicon vocal fold vibration pattern, as it is also visible in Table 4. Between 550 mm and 700 mm, the coupled mechanical-acoustic mode is aligned with the acoustic mode, which is the effect of the additional phonation-effective mode depicted in Table 4. In this regime, the acoustic back-coupling strongly influences the vibration frequency of the vocal fold motion. This indicates that acoustic effects of the fluid are essential for the realistic determination of the oscillation frequency. For longer ducts, the physical shape of the mode with index 9 is now the mode with index 10. Virtually shifting oscillation frequency of a fixed mode index 9 to a lower frequency (where mode 5 is aligned with the acoustic mode) compared to the uncoupled situation for a duct length below 550 mm due to the additional phonation-effective mode. In contrast to modes index 5 and index 9, mode index 6 does not couple at all to the acoustic mode because the oscillation pattern of the vocal folds is not acoustically effective (as indicated in the discussion of Table 4 by the counteracting the motion in *z*-direction). Regarding a more detailed analysis and the evolution of the modes with varying lengths, the modal assurance criterion (MAC) is evaluated for the simulated and measured modes.

The modal assurance criterion (MAC) is a measure of the similarity between different mechanical modes introduced in [33]. Given complex mode shapes in the form of two mode shape vectors $\Phi_{k}$ and $\Psi_{l}$, it computes as follows [33]

(13) $$MAC\left(\Phi_{k},\Psi_{l}\right)=\frac{{\left|\Phi_{k}^{T}\Psi_{l}^{*}\right|}^{2}}{\left(\Phi_{k}^{T}\Phi_{k}^{*}\right)\left(\Psi_{l}^{T}\Psi_{l}^{*}\right)}\;\;\cdot \;100.$$

Thereby, *k* and *l* are the indices of the respective modes, i.e., the *k*-th mode shape of Φ is compared with the *l*-th mode shape of Ψ. Hence, the MAC enables (i) comparisons of simulated mode shapes with measured mode shapes, (ii) comparisons of mode shapes originating from two different simulations, or (iii) self-comparisons of mode shapes from one simulation or one measurement (Auto-MAC) showing the self-similarity of different mode shapes of one configuration. However, the MAC requires that the mode shape pairs $\Phi_{k}$ and $\Psi_{l}$ are evaluated at identical coordinates. Therefore, the simulation result (i.e., the displacement field) is interpolated using the FE basis functions to the measurement points.

**Figure 10:**
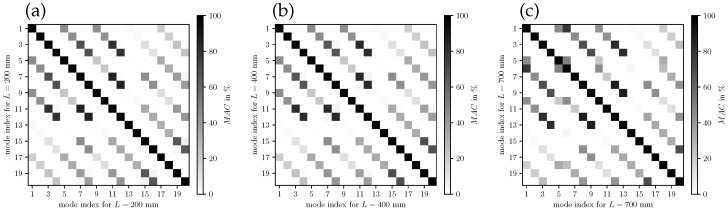
Auto-MAC of the simulation results. (**a**) Auto-MAC for L=200 mm, (**b**) Auto-MAC for L=400 mm, and (**c**) Auto-MAC for L=700 mm.

Firstly, the self-comparisons of mode shapes from one simulation as Auto-MAC is studied and illustrated in Figure 10. Therein, the MAC has been evaluated for the two LSV positions separately (see Figure 2), and the mean MAC values of the two measurement plane values are assessed. The three illustrations show the Auto-MAC of the simulated modes for the duct length 200 mm, 400 mm and 700 mm, in the three subfigures (a), (b), and (c), respectively. These lengths correspond to the three regimes (decoupling, alignment, shift) of mode 9 in Figure 9. As intended by the MAC, it shows a strong correlation of the modes with itself by the black squares in the diagonals of the matrix plots in Figure 10. For all individual modes, a low similarity is present concerning the other modes, which is expressed by the relatively low Auto-MAC values in the off-diagonal elements. This behavior is present in all three regimes investigated. Qualitatively, the three sub-figures look very similar, which expresses that the modes as a solution of the numerical system have persistent shapes with a length variation. Especially for length 200 mm and 400 mm and according to Table 4, the mode shapes are very similar (no coupling of the lower modes to the mechanic field) and therefore this is expected for the Auto-MAC values. This may allow us to use these shapes in a potential model order reduction over a wide range of operating points that are, respectively, length variations. From Figure 10, the mode indices and motions contributing to phonation show also correlations to each other, being the mode index numbers 1, 5, and 9 for length 200 mm and 400 mm. For length 700 mm, the phonation-effective mode (index 6) is present, showing that a high MAC value is present between mode index 1 and index 6.

**Figure 11:**
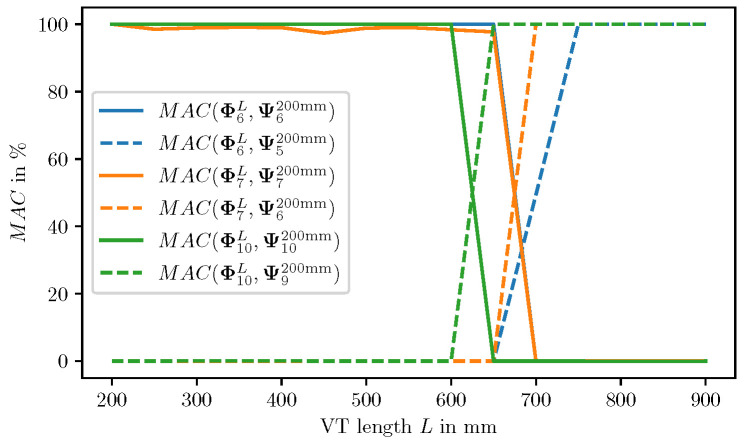
Comparison of MAC values for mode indices k∈{6,7,10} across VT length variations. The solid lines indicate the MAC based on the mode *k* of length 200 mm as reference, whereas the dashed lines indicate that the MAC is computed based on the mode k−1 as reference to illustrate a switch in the mode number.

In Figure 11, the MAC is depicted for the modes k∈{1,5,10}, along VT length variations using the simulation result data. With the *k*-th mode shape obtained from the VT length *L*, the MAC is computed for two reference mode shapes: (i) the *k*-th mode shape of L=200 mm denoted as $\Psi_{k}^{200\;mm}$, and (ii) the (k−1)-th mode shape for of L=200 mm denoted as $\Psi_{k-1}^{200\;mm}$. As depicted in Figure 11, a shift in mode indices is evident, i.e., for each mode index *k*, there is a critical VT length $L_{\mathrm{crit}}$ at which the $MAC(\Phi_{k}^{L},\Psi_{k}^{200\mathrm{mm}})$ switches from close to 100 to close to 0, and the $MAC(\Phi_{k}^{L},\Psi_{k-1}^{200\mathrm{mm}})$ simultaneously raises from close to 0 to close to 100. Therefrom it is evident that an index shift occurs, i.e., the *k*-th mode for $L<L_{\mathrm{crit}}$ is the (k−1)-th mode for $L>L_{\mathrm{crit}}$. Together with Figure 9 and Table 4, it can be concluded, that $L_{\mathrm{crit}}$ depends on the mode index *k*, and it is located at the intersection between the acoustic modes and the coupled acoustic-mechanic modes.

### 4.3. Comparison of Experimental and Numerical Results by Modal Assurance Criterion

To quantify the agreement between the measured surface velocities from the LSV measurements with the numerical simulations, the MAC is evaluated for the measured VT lengths. The result quantity of the simulation is the displacement $\vec{d}$, the measurement quantity of the LSV is the velocity $\dot{\vec{d}}=\left({\dot{d}}_{1},{\dot{d}}_{2},{\dot{d}}_{3}\right)=\partial \vec{d}/\partial t$. Furthermore, the simulation results are available in the frequency domain a priori, while the measurements are delivered in the time domain. Hence, the measured velocity field is Fourier transformed, such that a frequency resolution of Δf=10Hz is achieved. Then, the measured velocity field is compared to the simulation result (displacement) at the measurement points. The simulated displacement results are therefore multiplied by iω being the Fourier transform of ∂/∂t. Thereby, $\omega =2\pi f_{m}$ with $f_{m}$ being the mode frequency, respectively. The MAC matrices are depicted for both LSV positions depicted in Figure 2 separately.

**Figure 12:**
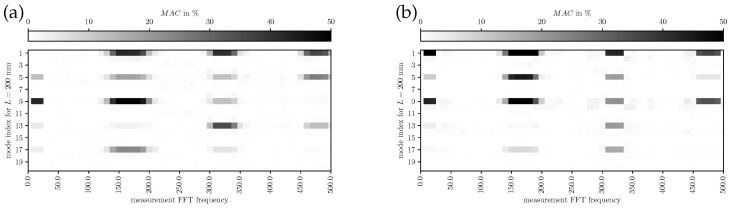
MAC for measured (LSV) and simulated modes with L=200 mm for (**a**) subglottal LSV Pos. 1 and (**b**) supraglottal LSV Pos. 2.

**Figure 13:**
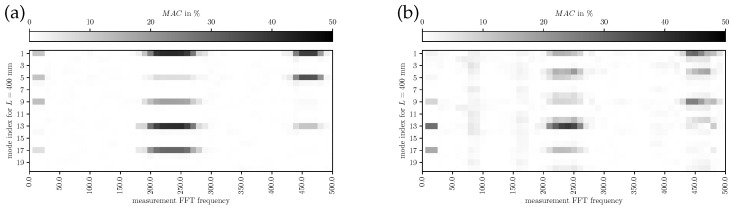
MAC for measured (LSV) and simulated modes with L=400 mm for (**a**) subglottal LSV Pos. 1 and (**b**) supraglottal LSV Pos. 2.

**Figure 14:**
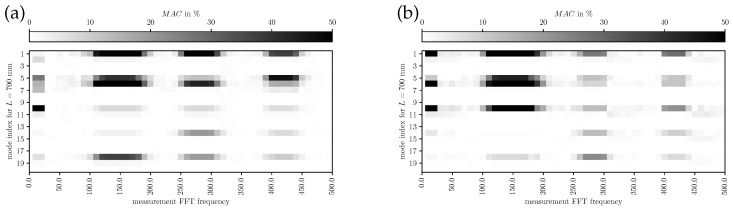
MAC for measured (LSV) and simulated modes with L=700 mm for (**a**) subglottal LSV Pos. 1 and (**b**) supraglottal LSV Pos. 2.

In Figure 12, Figure 13 and Figure 14, the MAC for L={200,400,700}mm is depicted, respectively. The modes identified by FE simulations are frequency-wise closely together; therefore, individual modes are hard to identify in the measurements. However, a qualitative assessment of mode shape morphology similarities is possible nevertheless. In Figure 12, it is visible that simulated modes 1 and 9 are mainly representing the experimental components at the measured fundamental frequency, providing evidence on the phonation effective modes in the decoupled regime. In Figure 13, the simulated mode 13 at 220 Hz explains the main characteristics of the experimental results, indicating a dominating mode shift. Furthermore, more modes add minor contributions to the overall vibration behavior compared to the decoupled regime. From Figure 14 one can conclude that the simulated modes 1, 5, and 6 (phonation-effective mode) are shifted to lower frequencies around 140Hz while also being similar to higher-frequency components at integer multiples (i.e., 280Hz and 420Hz). These comparisons to experimental modes provide a clear picture of which modes are essential for the phonation in the decoupled and coupled mechanical-acoustic regime.

## 5. Discussion

### 5.1. Model Limitations

Comparing Figure 5 and Figure 9 it can be clearly seen that the established model provides a clear condition when the acoustic-structure back-coupling is present. The frequency alignment and the decoupled regime can be predicted for varying duct length. The limitations of the linear eigenfrequency analysis are that the non-linear effects of the vocal fold contact and the multi-harmonic or chaotic oscillation behavior cannot be modeled accordingly. Therefore, the regimes (as indicated in Figure 5) “small shift” and “mode switching” cannot be explained by the coupled mechanical-acoustical model. Nevertheless, the model is useful to learn about the interaction of the mechanical and acoustical fields.

### 5.2. Oscillation Pattern and Relation to Other Voice Parameters

**Figure 15:**
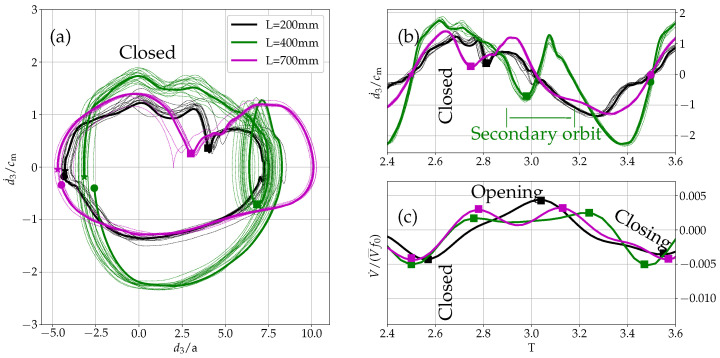
(**a**) Phase space diagram showing velocity ${\dot{d}}_{3}$ and $d_{3}$ displacement trajectories of the superior edge of the left vocal fold in the inferior-superior direction for all considered vocal tract lengths. The smaller orbit within the outer orbit (in the clockwise direction) for the L=400 mm case indicates periodic doubling and onset of chaotic behavior. The L=200 mm and L=700 mm cases show more regular orbits and with a pronounced notch for L=700 mm. The star marker (*) is the starting point and the circle marker (○) is the end point of one orbit. (**b**) Time history of the inferior-superior velocity ${\dot{d}}_{3}$ from PSV measurements. The square marker (■) indicates the periodic doubling in the L=400 mm case and the kink in the L=200 mm and L=700 mm case. (**c**) Time history of the flow rate derivative $\dot{V}\left(t\right)$ extracted from the PIV measurements of [31].

Initially, a harmonic oscillation pattern was assumed by the eigenvalue computation (see Equation (7)). In this paragraph, the limitations of this assumption are discussed using aspects of nonlinear dynamics, as introduced by Herzel et al. [34]. Figure 15 shows the phase space diagram of the oscillation in the *z*-direction (inferior-superior direction) of the left vocal fold’s superior edge (according to the coordinate system defined in Figure 7). Additionally, the time series of the velocity in *z*-direction ${\dot{d}}_{3}$, the time series of the flow derivative is displayed over one period. The velocity in *z*-direction ${\dot{d}}_{3}$ is measured at the point 7.5 mm and 7.1 mm (superior edge). Given the measured velocity ${\dot{d}}_{3}$, the displacement in *z*-direction $d_{3}$ is estimated by integration of the high-pass filtered (110 Hz) velocity signal ${\dot{d}}_{3}$. The initial conditions $d_{3}\left(t=0\right)=0$. In Figure 15, the displacement is normalized by the initial vocal fold gap a=0.2mm and the velocity by a typical mucosal wave speed of about $c_{m}=1\;m/s$ [35]. For normalized velocities ${\dot{d}}_{3}/c_{m}$ being plotted over the normalized displacement $d_{3}/a$, the phase space diagram orbits are turning in the clockwise direction for the left vocal fold (as indicated by the star as starting point and the circle as ending point marker). Additional markers for the second orbit and the notch in the magenta and black curve are marked by a square sign. The notch in the black and magenta curve is an inflection of the velocity, where the superior edge undergoes a wiggle-like motion going from deceleration to acceleration during the opening phase of the vocal fold. The orbit of $\left(d_{3}/a,{\dot{d}}_{3}/c_{m}\right)$ for the duct length 200 mm has a somewhat elliptical shape, with slight deviations from orbit to orbit. This can also be seen from the evolution of the ${\dot{d}}_{3}$ over a period of the oscillation. Despite some minor high-frequency patterns, the velocities time signal has a dominant harmonic content at the oscillation frequency $f_{0}$. According to Figure 6, the highest surface velocity corresponds to the phase of closed vocal folds and is indicated in Figure 15. Additionally, to the high-speed camera visualization, the glottal flow [31] was correlated to the time series of the velocity. The flow derivative is used to interpret the vocal fold motion, the opening and closing phase, and regarding [36], it shows a connection of intra-glottal vortices causing a more rapid closing resulting in an increased sound pressure level [37]. The flow rate derivative was estimated based on two-dimensional particle image velocimetry data [31] averaging ten lines perpendicular to the glottal jet at ten different streamwise positions starting at the vocal folds and assuming full coherence in the third dimension. In doing so, the time series of varying glottal flow rate $\dot{V}\left(t\right)$ was estimated and its time derivative dV(t)/dt computed. The estimated flow rate derivative was normalized by the mean glottal flow $\bar{V}$ and the fundamental frequency $f_{0}$. For the length 200 case, the flow derivative increases monotonically over half a period and declines approximately the other half of the period. This configuration is considered to be the base configuration and results in a sound pressure level (SPL) in front of the duct of 82.5 dB [31].

At a length of 400 mm the orbit shows a secondary loop indicating a periodic doubling at this length. A regular large orbit moves into a secondary orbit during each period. This is also visible in the velocity evolution over a period. The secondary orbit is indicated by the green square marker. It is indicated as secondary orbit and has nearly half the velocity amplitude of the dominating orbit. Variations from orbit to orbit are visible by the green dotted curves. Compared to the L=200 mm case, the orbit variations of the L=400 mm case are larger. In general, this indication of periodic doubling strongly violates the assumption of a harmonic ansatz, supporting the arguments that such a behavior cannot be explained by the eigenvalue simulation. It displays a multi-harmonic nature of the vocal fold oscillation at these conditions. The flow derivative for the length 400 case has a comparable small and extended positive part of the time series, spanning about 0.7*T* and a rapid negative closing dip twice as high as the positive part. This behavior of a sharp negative dip was also observed in the investigations conducted in [36]. Additionally, the flow derivative has a secondary oscillation which occurs at the same time as the periodic doubling. For this case, the SPL in front of the duct is 95 dB [31].

The orbit of $\left(z,{\dot{d}}_{3}\right)$ for the length L=700 mm has a elliptical shape with a kink in the positive part of the positive velocity fluctuations (indicated by a magenta square sign). Also for this case, slight deviations occur from orbit to orbit, which are comparable to the L=200 mm case. In this case, the positive part of the positive velocity fluctuations is oscillating, since it does stay positive no secondary orbit is formed. This is already a strong deviation from a pure harmonic velocity evolution. In connection to the closed vocal fold, the kink in the phase space diagram happens shortly after the vocal folds have closed. In this case, the flow derivative is positive for 0.6*T* and has a relative sharp negative dip compared to the length L=200 mm case. The evolution of the flow derivative looks very similar to the one from the length L=400 mm case. Where the kink occurs in the phase space diagram, the flow derivative shows a secondary oscillation. In this case, the SPL is 84 dB [31]. Comparing the three cases, it appears that the negative dip in the flow derivative is positively correlated with the SPL, as well as the inertance effect, as described by Titze [35], which gives a low supraglottal pressure that produces a push on vocal the vocal folds during closing. Due to the correlation with SPL, the vocal efficiency at the length L=400 mm case is about one order of magnitude higher than the length L=200 mm case [31]. Furthermore, the value of the vocal efficiency for the length L=700 mm case is increased [31], showing a positive correlation of the strength of the additional wiggle in the flow derivative and the vocal efficiency. In relation to Figure 8, the motion in the inferior-superior direction of the schematic and the phase space diagram are consistent.

## 6. Conclusions

The fluid-structure-acoustic interaction process, like human phonation process, is one of the most challenging physical phenomena. To enhance the understanding of this type of process, an experimental apparatus was designed to mechanically align the acoustic of the duct and the coupled mechanic-acoustic mode of the coupled vibroacoustic setup consisting of single mold silicone vocal folds and a straight duct. The experiments showed that when increasing the supraglottal duct of the apparatus, the acoustic eigenfrequency decreases monotonically. In the case when the acoustic eigenfrequency of the duct came into the range of the fundamental (mechanical) vibration frequency of the silicone vocal folds their vibration frequency deviated from it. This effect is dominant and strong deviations occur, when the acoustic eigenfrequency of the duct is lower than the corresponding mechanical eigenfrequency.

Regarding these experimental findings, the vocal folds motion of the uncoupled and the coupled mechanical-acoustic eigenvalue problem are investigated. The purpose of the simulation is to show that for a length smaller than the critical length (crossing of the acoustic and the mechanical eigenfrequency), the mechanical eigenmodes of the vocal folds in the neighborhood of the fundamental frequency are not influenced by the acoustic (compressible) subsystem. Whereas, for a length longer than the critical length, the combined system is of importance and the vibration frequency of the vocal folds is aligned with the acoustic mode frequency. The results demonstrate that changing the vocal tract length has an influence on the frequency of the mode arising by the coupled mechanic-acoustic field. Furthermore, the quantitative comparison between numerical and experimental results by means of the MAC exhibits a strong correlation of the coupled mechanic-acoustic mode, indicating a strong contribution of this mode on phonation. It was found that a changing vocal tract length allows for a changing frequency of the coupled mode that greatly contributes to phonation. As a consequence of this analysis, the findings report the importance of the interaction and the back-coupling of the acoustic onto the mechanical structure in certain regimes. Whereas under normal conditions, the back-coupling can be neglected as reported in numerous studies before. Finally, the use of the eigenmode analysis is an elegant way of investigating the dependence of the modes on each other. This may allow us to use these shapes in a potential model order reduction over a wide range of operating points that are, respectively, length variations. Relating to the acoustic-structure interaction, one recent publication by Manconi et al. [38] included the characterization of this interaction by a dispersion curve. This approach could be transferred to human phonation to display the dispersion relation of the mucosal wave, but, as a prerequisite, equally spaced surface displacement data is necessary. However, the measurement data capturing all nonlinear effects are not equally spaced. Hence, the findings presented in [38] provide an interesting approach for future investigations.

Finally, the MAC measure between the simulated and experimental modes showed which mode shapes effectively contribute to the phonation and which modes do not contribute. As numerical and experimental results are in good agreement, the model can be used to provide explanatory insight for acoustic contributions of individual modes. This analysis provided a clear picture of both the coupled (source-filter interaction) and the decoupled (normal phonation) mechanical-acoustic regime. Furthermore, the results showed strong correlations of the obtained vocal fold motion characteristics with previously found correlations to other voice parameters like vocal efficiency and the sound pressure level.

## Figures and Tables

**Table 1:** Model parameters for frequency-dependent Young’s Modulus E(f) determined by Rupitsch et al. (“mixture 113”) [18].

$A_{R}$	$B_{R}$	$C_{R}$	$A_{I}$	$B_{I}$	$C_{I}$	ν
7.02 kPa	$1.09\times 10^{1}$	$8.02\times 10^{2}$	$4.05\times 10^{3}$	$1.07\times 10^{1}$	$-1.21\times 10^{3}$	0.499

**Table 2:** Rayleigh parameters α and β for the operating point $f_{1}=140\mathrm{Hz}$ as well as Young’s modulus evaluated by approximating the dynamic model of Rupitsch [18].

$f_{1}$	$\alpha (f_{1})$	$\beta (f_{1})$	$E_{R}\left(f_{1}\right)$
140 Hz	126.2313	$1.631\times 10^{-4}$	10.26 kPa

**Table 3:** Mesh convergence analysis of the numerical model for the coupled mechanic-acoustic system. The supraglottal length *L* was 1050 mm for all meshes (see Figure 3).

Mesh	Approx. Elem. Size (in mm)			Wavelength at *f* = 8.5 kHz		${\mathrm{Err}}_{\mathrm{rel},f}^{L_{2}}$
	Duct	VT		Duct	VT	
mesh 1	4.0	1.4		λ/10	λ/29	0.0086
mesh 2	2.0	0.7		λ/20	λ/58	0.0020
mesh 3 (reference)	1.0	0.35		λ/40	λ/115	—

**Table 4:** Numerically obtained mode shapes for different VT lengths *L*. The color indicates the displacement component $d_{3}$ in *z*-direction, while the *x* and *y*-components of the displacement are visualized by a scaled geometry deformation. Negative Displacement $d_{3}<0$ is colored blue, positive displacement $d_{3}>0$ is colored red, and $d_{3}=0$ is colored green. The displacement component $d_{3}$ is normalized to the maximum absolute value.

VT Length	Mode Index 1	Mode Index 2	Mode Index 3	Mode Index 4	Mode Index 5
L=200 mm	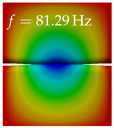	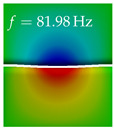	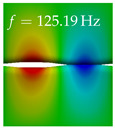	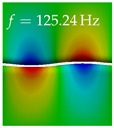	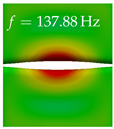
L=400 mm	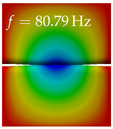	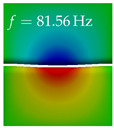	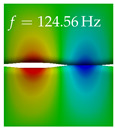	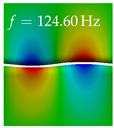	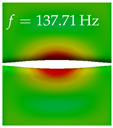
L=700 mm	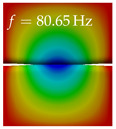	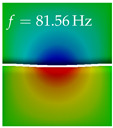	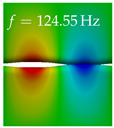	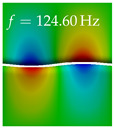	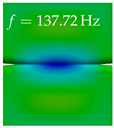
L=800 mm	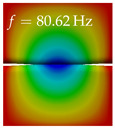	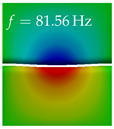	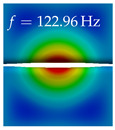	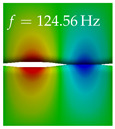	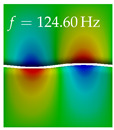
**VT Length**	**Mode Index 6**	**Mode Index 7**	**Mode Index 8**	**Mode Index 9**	**Mode Index 10**
L=200 mm	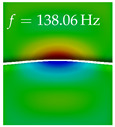	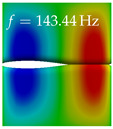	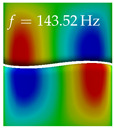	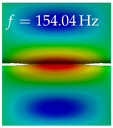	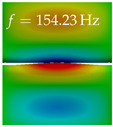
L=400 mm	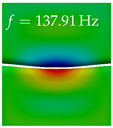	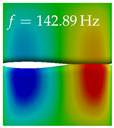	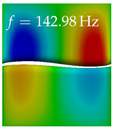	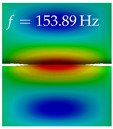	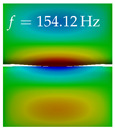
L=700 mm	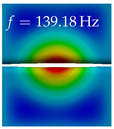	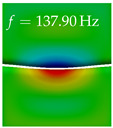	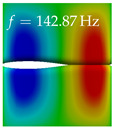	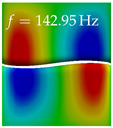	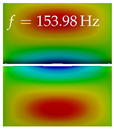
L=800 mm	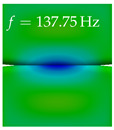	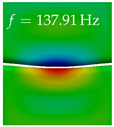	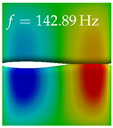	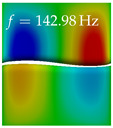	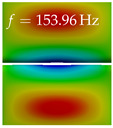

## Data Availability

The data presented in this study are available on request from the corresponding author.

## References

[1] Döllinger M., Zhang Z., Schoder S., Šidlof P., Tur B., Kniesburges S. (2023). Overview on state-of-the-art numerical modeling of the phonation process. Acta Acust..

[2] Schoder S., Maurerlehner P., Wurzinger A., Hauser A., Falk S., Kniesburges S., Döllinger M., Kaltenbacher M. (2021). Aeroacoustic sound source characterization of the human voice production-perturbed convective wave equation. Appl. Sci..

[3] Maurerlehner P., Schoder S., Freidhager C., Wurzinger A., Hauser A., Kraxberger F., Falk S., Kniesburges S., Echternach M., Döllinger M. (2021). Efficient numerical simulation of the human voice: simVoice—A three-dimensional simulation model based on a hybrid aeroacoustic approach. e i Elektrotech. Informationstechnik.

[4] Schoder S., Kraxberger F., Falk S., Wurzinger A., Roppert K., Kniesburges S., Döllinger M., Kaltenbacher M. (2022). Error detection and filtering of incompressible flow simulations for aeroacoustic predictions of human voice. J. Acoust. Soc. Am..

[5] Zhang Z. (2016). Cause-effect relationship between vocal fold physiology and voice production in a three-dimensional phonation model. J. Acoust. Soc. Am..

[6] Fant G. (1971). Acoustic Theory of Speech Production: With Calculations Based on X-ray Studies of Russian Articulations.

[7] Titze I.R. (2008). Nonlinear source–filter coupling in phonation: Theory. J. Acoust. Soc. Am..

[8] Sundberg J., Lã F., Granqvist S. (2023). Fundamental frequency disturbances in female and male singers’ pitch glides through long tube with varied resistances. J. Acoust. Soc. Am..

[9] Lucero J.C., Lourenço K.G., Hermant N., Van Hirtum A., Pelorson X. (2012). Effect of source–tract acoustical coupling on the oscillation onset of the vocal folds. J. Acoust. Soc. Am..

[10] Zhang Z., Neubauer J., Berry D.A. (2006). Aerodynamically and acoustically driven modes of vibration in a physical model of the vocal folds. J. Acoust. Soc. Am..

[11] Zhang Z., Neubauer J., Berry D.A. (2009). Influence of vocal fold stiffness and acoustic loading on flow-induced vibration of a single-layer vocal fold model. J. Sound Vib..

[12] Echternach M., Herbst C.T., Köberlein M., Story B., Döllinger M., Gellrich D. (2021). Are source-filter interactions detectable in classical singing during vowel glides?. J. Acoust. Soc. Am..

[13] Migimatsu K., Tokuda I.T. (2019). Experimental study on nonlinear source–filter interaction using synthetic vocal fold models. J. Acoust. Soc. Am..

[14] Näger C., Lodermeyer A., Becker S. Charakterisierung der Stimmlippenvibration an einem synthetischen Larynx-Modell mittels Laser-Scanning-Vibrometrie. Proceedings of the DAGA 2022.

[15] Näger C., Kniesburges S., Becker S. Investigation of Acoustic Back-coupling in Human Phonation via Particle Image Velocimetry. Proceedings of the Forum Acusticum 2023.

[16] Falk S., Kniesburges S., Schoder S., Jakubaß B., Maurerlehner P., Echternach M., Kaltenbacher M., Döllinger M. (2021). 3D-FV-FE aeroacoustic larynx model for investigation of functional based voice disorders. Front. Physiol..

[17] Scherer R.C., Shinwari D., De Witt K.J., Zhang C., Kucinschi B.R., Afjeh A.A. (2001). Intraglottal pressure profiles for a symmetric and oblique glottis with a divergence angle of 10 degrees. J. Acoust. Soc. Am..

[18] Rupitsch S.J., Ilg J., Sutor A., Lerch R., Döllinger M. (2011). Simulation based estimation of dynamic mechanical properties for viscoelastic materials used for vocal fold models. J. Sound Vib..

[19] Durst F., Heim U., Ünsal B., Kullik G. (2003). Mass flow rate control system for time-dependent laminar and turbulent flow investigations. Meas. Sci. Technol..

[20] Lodermeyer A., Becker S., Döllinger M., Kniesburges S. (2015). Phase-locked flow field analysis in a synthetic human larynx model. Exp. Fluids.

[21] Lodermeyer A., Bagheri E., Kniesburges S., Näger C., Probst J., Döllinger M., Becker S. (2021). The mechanisms of harmonic sound generation during phonation: A multi-modal measurement-based approach. J. Acoust. Soc. Am..

[22] ANSYS, Inc. (2022). Ansys Mechanical, Release 2022 R2.

[23] Kohnke P., ANSYS, Inc. (2009). Theory Reference for the Mechanical APDL and Mechanical Applications.

[24] Kaltenbacher M. (2015). Numerical Simulation of Mechatronic Sensors and Actuators.

[25] Dabbaghchian S., Arnela M., Engwall O., Guasch O. (2021). Simulation of vowel-vowel utterances using a 3D biomechanical-acoustic model. Int. J. Numer. Methods Biomed. Eng..

[26] Story B.H., Laukkanen A.M., Titze I.R. (2000). Acoustic impedance of an artificially lengthened and constricted vocal tract. J. Voice.

[27] Murray P.R., Thomson S.L. (2012). Vibratory responses of synthetic, self-oscillating vocal fold models. J. Acoust. Soc. Am..

[28] Kraxberger F., Museljic E., Kurz E., Toth F., Kaltenbacher M., Schoder S. The Nonlinear Eigenfrequency Problem of Room Acoustics with Porous Edge Absorbers. Proceedings of the Forum Acusticum 2023, European Acoustics Association.

[29] Kraxberger F., Kurz E., Weselak W., Kubin G., Kaltenbacher M., Schoder S. (2023). A Validated Finite Element Model for Room Acoustic Treatments with Edge Absorbers. Acta Acust..

[30] Sondhi M., Schroeter J. (1987). A hybrid time-frequency domain articulatory speech synthesizer. IEEE Trans. Acoust. Speech Signal Process..

[31] Näger C., Kniesburges S., Tur B., Schoder S., Becker S. (2023). Investigation of Acoustic Back-coupling in Human Phonation on a Synthetic Larynx Model. Bioengineering.

[32] Titze I.R., Martin D.W. (1998). Principles of Voice Production.

[33] Pastor M., Binda M., Harčarik T. (2012). Modal assurance criterion. Procedia Eng..

[34] Herzel H., Berry D., Titze I.R., Saleh M. (1994). Analysis of vocal disorders with methods from nonlinear dynamics. J. Speech Lang. Hear. Res..

[35] Titze I.R. (2022). How can vocal folds oscillate with a limited mucosal wave?. JASA Express Lett..

[36] Sundström E., Oren L., de Luzan C.F., Gutmark E., Khosla S. Fluid-Structure Interaction Analysis of Aerodynamic and Elasticity Forces During Vocal Fold Vibration. J. Voice.

[37] Titze I.R. (2006). Theoretical analysis of maximum flow declination rate versus maximum area declination rate in phonation. J. Speech Lang. Hear. Res..

[38] Manconi E., Mace B.R., Garziera R. (2023). Wave Propagation in Laminated Cylinders with Internal Fluid and Residual Stress. Appl. Sci..

